# Effectiveness and Safety of Micro‐Plasma Radiofrequency Treatment Combined With Autologous Chyle Fat Grafting Treatment for Hypertrophic Scars: A Retrospective Study

**DOI:** 10.1111/jocd.16728

**Published:** 2024-12-27

**Authors:** Peixuan Zhang, Haina Pei, Guiwen Zhou, Qiang Fu, Ruiqi Bai, Pianpian Lin, Qian Wu, Xiao Xu, Minliang Chen

**Affiliations:** ^1^ Department of Plastic and Reconstructive Surgery, Senior Department of Burns and Plastic Surgery The Fourth Medical Center of Chinese PLA General Hospital Beijing China; ^2^ Department of Burn and Plastic Surgery Hainan Hospital of Chinese PLA General Hospital Sanya Hainan China; ^3^ Department of Ophthalmology The Third Medical Center of Chinese PLA General Hospital Beijing China

**Keywords:** chyle fat, hypertrophic scar, micro‐plasma radiofrequency, retrospective study

## Abstract

**Background:**

Hypertrophic scar (HS) is a fibroproliferative disorder resulting from abnormal healing of skin tissue after injury. Although various therapies are currently employed in clinical to treat HSs, there is no widely accepted standard therapy. Micro‐plasma radiofrequency (MPR) and autologous chyle fat grafting are emerging treatments for this condition, and they have demonstrated promising therapeutic outcomes in clinical applications. The aim of this study is to investigate the effectiveness and safety of combining MPR with autologous chyle fat grafting for the treatment of HSs.

**Methods:**

We performed a retrospective study on patients diagnosed with HS in a single center between January 2020 and December 2023. According to the treatments, patients were divided into three groups, with 6 months follow‐up. The single therapy group received MPR alone for two times. The combined therapy Group 1 first received the MPR treatment followed by the combined treatment. The combined therapy Group 2 first received the combined treatment and then received the MPR treatment. The effectiveness of treatment was evaluated using the Vancouver Scar Scale (VSS) and the Patient Scar Assessment Scale (PSAS). The Visual Analog Scale (VAS) was used to assess the patients' pain on the day of treatment and 1 day after treatment. Adverse events and complications were recorded to assess the safety of treatment.

**Results:**

A total of 73 patients diagnosed with HS were enrolled in this study, including 35 patients in the single therapy group, 18 patients in the combined therapy Group 1, and 20 patients in the combined therapy Group 2. After the treatments were completed, all three groups exhibited significant effectiveness. The two combined therapy groups scored lower after treatments in the VSS, which includes height, vascularity, pliability, and total scores, as well as in the PSAS, which includes color, stiffness, thickness, and total scores, compared to the single therapy group, with a statistically significant difference. Regarding pain response to treatment, there was no statistical difference in VAS among the three groups. No statistical difference in the overall incidence of adverse events was observed among the three groups, and no severe complications were recorded.

**Conclusions:**

This study revealed the combination of MPR and autologous chyle fat grafting showed superior effectiveness compared to MPR alone in treating HSs, without any observed increase in overall adverse event frequency. For patients diagnosed with HS, this combination therapy stands as a promising and effective clinical intervention.

**Abbreviations**
ADSCs: adipose‐derived stem cells
ECM: extracellular matrix
HGF: hepatocyte growth factor
HS: hypertrophic scar
IL: interleukin
MCP‐1: macrophage chemotactic protein‐1
MMPs: metalloproteinases
MPR: micro‐plasma radiofrequency
Nrf2: nuclear factor erythroid‐2‐related factor 2
ROS: reactive oxygen species
Smad: small mothers against decapentaplegic
TGF‐β: transforming growth factor‐β
TIMPs: tissue inhibitor of metalloproteinases
TNF‐α: tumor necrosis factor‐α
VAS: Visual Analog Scale
VSS: Vancouver Scar Scale
α‐SMA: α‐smooth muscle actin

## 1 Introduction

The skin, being the largest organ of the human body, functions as a protective barrier against external environmental changes. Various factors such as trauma, burns, infection, and surgery can result in skin injuries. The process of wound healing is complex, including five stages: hemostasis, inflammation, proliferation, re‐epithelialization, and remodeling [1]. Hypertrophic scar (HS) is a fibroproliferative disease that arises from abnormal wound healing following dermal tissue injury. It is characterized by ongoing local inflammation and excessive deposition of collagen [2, 3]. HSs not only impact aesthetic appearance but also impose significant psychological burdens on affected individuals. The management of HSs can be classified into two categories: conservative and surgical therapies. Conservative management strategies encompass a variety of therapies, including compression therapy, the use of gel sheet, corticosteroid injection, and laser treatment. The surgical excision of scar tissue is an intervention to alleviate or address the functional impairments resulting from scar contracture [4]. However, each treatment has different side effects that need to be addressed. Currently, no universally accepted standard therapy exists for the management of HSs, posing a tremendous challenge for both clinicians and patients in terms of treatment and prevention.

In recent years, with the increasing application of autologous fat grafting and adipose‐derived stem cells (ADSCs) in regenerative medicine, many researchers have applied them to the treatment of HSs. Due to the dense fibrous tissue composition in HSs, injecting granular fat into them can be challenging. Tonnard et al. proposed the concept of nanofat and observed that its ability to restore tissue volume was significantly limited in clinical practice. However, nanofat showed greater promise in the fields of skin rejuvenation and scar treatment [5]. Nanofat has been used to treat atrophic scars, skin wrinkles, and skin discoloration, while there is a paucity of research focused on its application in the treatment of HSs [6, 7]. In a previous study, Xu et al. demonstrated the remarkable therapeutic effectiveness of autologous chyle fat grafting in 80 patients with HS. Histological analysis revealed a decrease in dermis thickness, fibroblast density and quantity within the HSs. Additionally, a decline in blood vessel density was observed, while the arrangement, quantity, and shape of collagen III exhibited normalization [8].

Micro‐plasma radiofrequency (MPR) has emerged as a promising technique for scar management for the past few years. Several studies have demonstrated the effectiveness of MPR in treating HSs [9]. The principle of MPR for the treatment of HSs is mild epidermal ablation and the generation of a thermal effect within the deep dermis. Unipolar radiofrequency excites nitrogen molecules, generating micro‐plasma that induces micro‐sparks on the skin surface. These sparks result in mild epidermal ablation and create micro‐channels that perforate the dermal surface. The thermal energy produced by radiofrequency is uniformly distributed within the deep collagen tissue, inducing the generation of new collagen and dermal remodeling, and ultimately achieving treatment goals [10, 11]. MPR meets the need of minimally invasive and safe technology for scar treatment.

Recent research has demonstrated a growing interest in the synergistic use of multiple therapies for the treatment of HSs. This retrospective study aimed to investigate the effectiveness and safety of autologous chyle fat grafting combined with MPR in the clinical treatment of HSs, thus proposing a novel and potentially effective combined treatment approach for HSs.

## 2 Methods

### 2.1 Patient Selection

We performed a single‐center, retrospective study on patients diagnosed with HS in the Department of Burn and Plastic Surgery of our hospital, between January 2020 and December 2023. The study was approved by the ethics committee of our hospital (No. 2023KY127‐HS001) and was conducted in accordance with the Declaration of Helsinki.

The inclusion criteria for the patients in this study include: (1) clinically diagnosed with HS; (2) scar formation time more than 1 month; (3) receiving either autologous chyle fat grafting combined with MPR treatment or MPR treatment alone; (4) with complete data available. However, patients who had previously undergone other treatments for HS in 6 months before receiving treatment were excluded from this study.

All patients received two treatments, and they were divided into three groups based on the method and order of treatment they received: the first group of patients received two MPR treatments, referred to as the single therapy group; the second group of patients first received an MPR treatment followed by the combined treatment, referred to as the combined therapy Group 1; the third group of patients first received the combined treatment and then received an MPR treatment, referred to as the combined therapy Group 2. In the combined treatment, the patients first received MPR treatment followed by autologous chyle fat grafting. The interval between the two treatments was 1–3 months. Demographic data, including gender, age, scar formation time, scar area, scar etiology, and scar location were collected for all patients.

### 2.2 MPR Treatment

Before treatment, lidocaine cream was applied locally to the patient's treatment area. The power of the fractional MPR (Accent, Alma Lasers, Caesarea, Israel) was set to 60–90 W depending on the patient's condition. The same operator scrolled the roller head in horizontal, vertical, and diagonal direction at the same speed for two times until an even distribution of yellow spots appeared.

### 2.3 Autologous Chyle Fat Grafting Treatment

**FIGURE 1:**
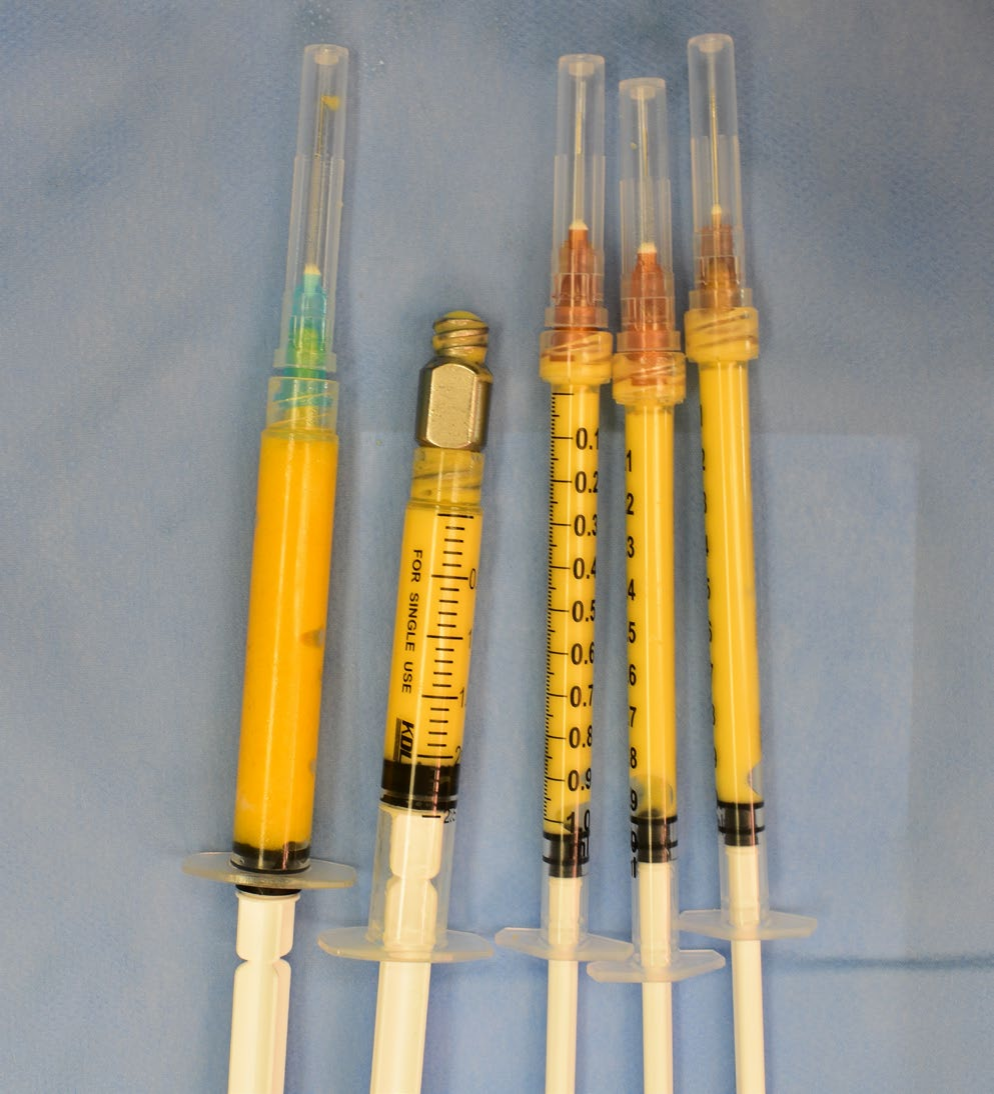
Autologous chyle fat.

Infiltration solution (ringer's solution 500 mL + 1% lidocaine 15 mL + 0.1% epinephrine 1.3 mL) was prepared. According to the preoperative marking of the liposuction area, infiltration solution was injected into the subcutaneous fat layer of the marked area. The process of fat harvesting was carried out from the thighs and abdomen, using a 20‐mL syringe with a pumpback of 10 mL (with a negative pressure of approximately −60 kPa). Coleman's technique was used to prepare the fat implant [12]. The harvested fat was filtered, cleaned, purified with Ringer's solution, and removed coarse fibrous tissue. The fat was transferred 20 times between two interconnected syringes using a 1.2‐mm nanometer transverter and then transferred 10 times using a 0.8‐mm nanometer transverter to prepare chyle fat which exhibits a whitish appearance (Figure 1). Subsequently, the subcutaneous portion of the infusion solution was extruded, and a sterile gauze bandage was applied to the treated area.

After a 15‐min waiting period, a 0.5 cm incision was made at a hidden site on one end of the scar. The prepared chyle fat was then directly injected into the scar through a 2 mL syringe during the process of needle withdrawal. The incision was subsequently sutured with 6–0 nylon thread. The adjacent area was compressed with the dressing, ensuring no compression of the scar.

### 2.4 Assessments of Effectiveness and Safety

The follow‐up of patients continued until 6 months after the completion of treatments. The effectiveness of treatment was evaluated using the Vancouver Scar Scale (VSS) and the Patient Scar Assessment Scale (PSAS) [13, 14]. The VSS of patients were assessed by two professionally trained physicians who were blinded to the study design before and 6 months after treatments. The total score of VSS is 15, including pigmentation (0–3 points), vascularity (0–3 points), height (0–4 points), and pliability (0–5 points). Based on the previous literature, we classified the extent of VSS improvement into four grades: 0%–25%, 26%–50%, 51%–75%, and 76%–100% [15]. The PSAS were self‐assessed by the patients before and 6 months after treatments, it consists of six items: pain, itch, color, stiffness, thickness, and irregularity. Each item is scored from 1 to 10 for a total score of 60. Higher scores indicate more severe scar.

The Visual Analog Scale (VAS) was used to assess the patients' pain on the day of treatment and 1 day after treatment. The single therapy group was assessed after the first treatment, whereas the combination therapy Groups 1 and 2 were assessed after receiving the combined treatment. The VAS is scored on a scale of 0–10, with higher scores indicating more severe pain.

Patient satisfaction was systematically gathered and it was classified into three categories: satisfied, generally satisfied, and dissatisfied. The patient satisfaction rate is equal to the percentage of the sum of the numbers of satisfied and generally satisfied patients out of the total number of patients.

Adverse events such as erythema, pruritus, swelling, and ecchymosis, as well as complications such as infection and necrosis, were recorded to assess the safety of the treatment.

### 2.5 Statistical Analysis

SPSS 25.0 was used for statistical analysis in this study. Quantitative data were expressed as mean ± standard deviation or median (quartile), qualitative data were expressed as numbers and percentages. Analysis of Variance, Kruskal–Wallis *H* test, chi‐square (*χ* ^2^) test, and Wilcoxon signed‐rank test were used for inter‐group comparisons or intra‐group comparisons among three groups. *p* ≤ 0.05 indicates a statistically significant difference.

## 3 Results

A total of 73 patients diagnosed with HS were included in this study. Among them, there were 35 patients in the single therapy group, 18 patients in the combined therapy Group 1, and 20 patients in the combined therapy Group 2 (Table 1). The proportion of males was higher in the combined therapy Group 1 compared to the other two groups. The median age of patients in the combined therapy Group 2 was approximately 31 years, while the other two groups were 23 years. The etiology of scars included burn, scald, trauma and surgery. The median time of scar formation was 4 months in the single therapy group, compared to 6.5 and 7 months in the two combined therapy groups. In all three groups, the median scar area did not exceed 10 square centimeters and the head and neck were the most common location of scars. Overall, there was no significant difference in demographic characteristics among the three groups.

**TABLE 1:** Demographic characteristics of patients.

Variables	Single therapy group (*n* = 35)	Combined therapy Group 1 (*n* = 18)	Combined therapy Group 2 (*n* = 20)	Statistical variables	*p* value
Gender, *n* (%)				*χ* ^2^ = 4.333	0.115
Male	19 (54.3)	11 (61.1)	6 (30.0)		
Female	16 (45.7)	7 (38.9)	14 (70.0)		
Age, years	23.0 ± 13.9	23.3 ± 10.9	31.3 ± 16.0	*F* = 2.537	0.086
Time of scars, months	4.0 (3.0, 12.0)	6.5 (1.8, 21.0)	7.0 (5.0, 46.5)	*H* = 5.082	0.079
Area of scars, cm^2^	8.0 (5.0, 12.0)	8.0 (5.8, 12.8)	10.0 (5.0, 18.8)	*H* = 0.651	0.722
Etiology of scars, *n* (%)				*χ* ^2^ = 2.847	0.850
Burn	9 (25.7)	4 (22.2)	3 (15.0)		
Scald	6 (17.1)	4 (22.2)	4 (20.0)		
Trauma	12 (34.3)	6 (33.3)	5 (25.0)		
Surgery	8 (22.9)	4 (22.2)	8 (40.0)		
Location of scars, *n* (%)				*χ* ^2^ = 2.764	0.620
Head and neck	20 (57.1)	13 (72.2)	11 (55.0)		
Trunk	8 (22.9)	3 (16.7)	7 (35.0)		
Limbs	7 (20.0)	2 (11.1)	2 (10.0)		

**FIGURE 2:**
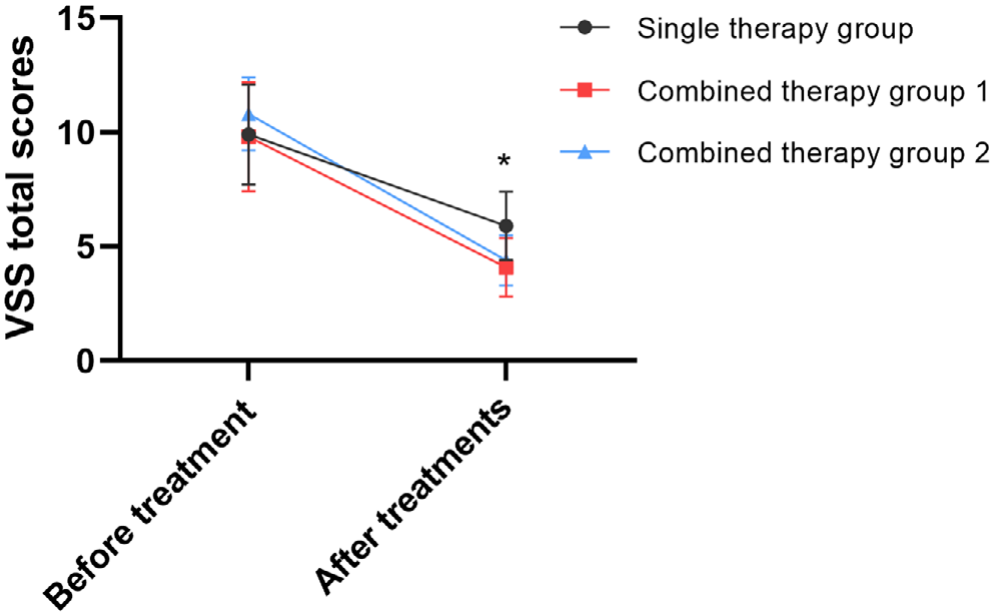
Line chart of the VSS total scores. *There is a statistically significant difference among the three groups (*p* ≤ 0.05).

Before treatment, no significant difference was observed among the three groups in terms of pigmentation, height, vascularity, pliability, and VSS total scores. Following two treatments, a statistically significant decrease was noted in the VSS total scores and all sub‐scores within all three groups (Table 2). However, the two combined therapy group demonstrated markedly superior effectiveness when compared to the single therapy group (Figure 2). Specifically, the height (*p* = 0.050), vascularity (*p* = 0.024), pliability (*p* = 0.009), and total scores (*p* < 0.001) in the combined therapy Group 1 were significantly lower than those in the single therapy group, whereas the height (*p* = 0.010), flexibility (*p* = 0.029), and total scores (*p* = 0.001) in the combined therapy Group 2 were significantly lower than those in the single therapy group. However, no statistical difference was found between the two combined therapy groups. Regarding the extent of VSS improvement, both combined therapy groups showed a significantly greater improvement compared to the single therapy group (*p* < 0.001), with over 80% of patients experiencing an improvement of over 50% (Table 3).

**TABLE 2:** VSS sub‐scores and total scores before and after treatments.

	Single therapy group	Combined therapy Group 1	Combined therapy Group 2	Statistical variables	*p* value^b^
Pigmentation					
Before treatment	2.0 (2.0, 3.0)	2.5 (2.0, 3.0)	3.0 (2.0, 3.0)	*H* = 2.225	0.329
After treatments	1.0 (1.0, 2.0)	1.0 (1.0, 1.0)	1.0 (1.0, 1.0)	*H* = 5.193	0.075
*p* value^a^	< 0.001	< 0.001	< 0.001		
Height					
Before treatment	2.0 (2.0, 3.0)	2.5 (2.0, 3.0)	3.0 (2.0, 3.0)	*H* = 0.837	0.658
After treatments	1.0 (1.0, 2.0)	1.0 (1.0, 1.0)*	1.0 (0.0, 1.0)*	*H* = 10.698	0.005
*p* value^a^	< 0.001	< 0.001	< 0.001		
Vascularity					
Before treatment	2.0 (2.0, 2.0)	2.0 (1.8, 2.0)	2.0 (2.0, 3.0)	*H* = 3.153	0.207
After treatments	1.0 (1.0, 2.0)	1.0 (0.8, 1.0)*	1.0 (1.0, 1.0)	*H* = 7.674	0.022
*p* value^a^	< 0.001	< 0.001	< 0.001		
Pliability					
Before treatment	3.0 (2.0, 3.0)	3.0 (2.0, 3.0)	3.0 (3.0, 4.0)	*H* = 2.559	0.278
After treatments	2.0 (1.0, 2.0)	1.0 (1.0, 2.0)*	1.0 (1.0, 2.0)*	*H* = 11.481	0.003
*p* value^a^	< 0.001	< 0.001	< 0.001		
Total					
Before treatment	9.9 ± 2.2	9.8 ± 2.4	10.8 ± 1.6	*F* = 1.489	0.233
After treatments	5.9 ± 1.5	4.1 ± 1.3*	4.4 ± 1.1*	*F* = 12.915	< 0.001
*p* value^a^	< 0.001	< 0.001	< 0.001		

Abbreviation: VSS, Vancouver Scar Scale.

* Compared to the single therapy group, *p* ≤ 0.05.

^a^ Comparison between the scores of the VSS before and after treatments.

^b^ Comparison between the scores of the VSS in different groups.

**TABLE 3:** Extent of VSS improvement.

	Single therapy group, *n* (%)	Combined therapy Group 1, *n* (%)	Combined therapy Group 2, *n* (%)	Statistical variables	*p* value
Percentage				*H* = 38.333	< 0.001
0%–25%	4 (11.4)	0 (0)	0 (0)		
26%–50%	27 (77.1)	2 (11.1)	4 (20.0)		
51%–75%	4 (11.4)	16 (88.9)	16 (80.0)		
76%–100%	0 (0)	0 (0)	0 (0)		

Abbreviation: VSS, Vancouver Scar Scale.

**FIGURE 3:**
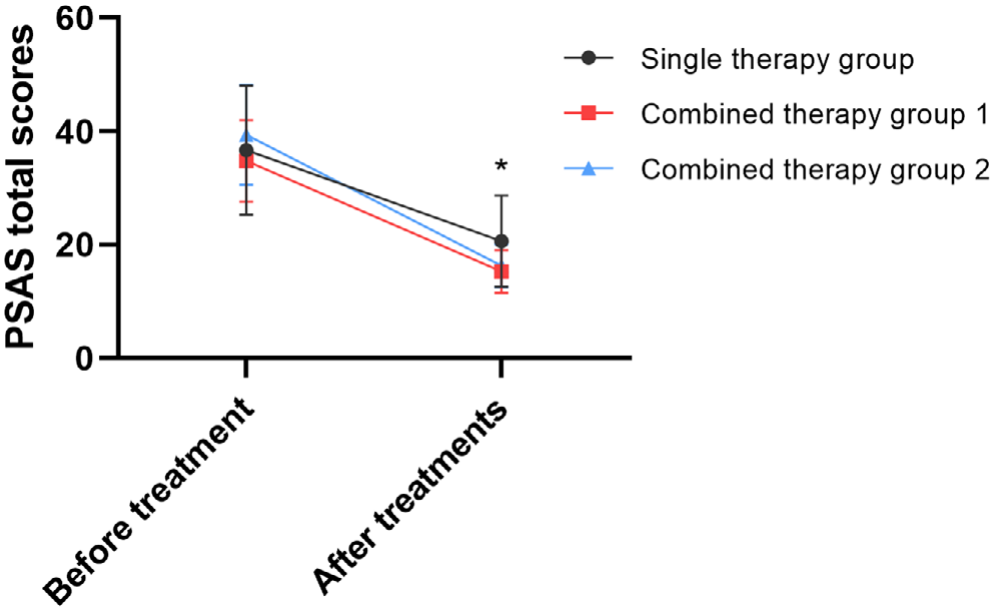
Line chart of the PSAS total scores. *There is a statistically significant difference among the three groups (*p* ≤ 0.05).

The three groups showed no significant difference in the six sub‐scores of the PSAS before treatment, as well as in the total scores. A statistically significant decrease in all scores of the three groups was observed after treatments (Table 4). Similar to the results of VSS, there were statistical differences between the two combined therapy groups and the single therapy group, but no statistical difference between the two combined therapy groups (Figure 3). Color (*p* = 0.015), stiffness (*p* = 0.016), thickness (*p* = 0.041), and total scores (*p* = 0.015) of the combined therapy Group 1 were significantly lower than those of the single therapy group, as were the stiffness (*p* = 0.043), thickness (*p* = 0.006) and total scores (*p* = 0.048) of the combined therapy Group 2.

**TABLE 4:** PSAS sub‐scores and total scores before and after treatments.

	Single therapy group	Combined therapy Group 1	Combined therapy Group 2	Statistical variables	*p* value^b^
Pain					
Before treatment	5.0 (3.0, 7.0)	3.5 (2.0, 5.0)	4.0 (3.0, 5.8)	*H* = 4.960	0.084
After treatments	2.0 (1.0, 3.0)	1.0 (1.0, 2.0)	2.0 (1.0, 2.0)	*H* = 5.268	0.072
*p* value^a^	< 0.001	< 0.001	< 0.001		
Itch					
Before treatment	5.0 (4.0, 6.0)	4.5 (3.8, 5.0)	5.0 (4.0, 6.8)	*H* = 1.854	0.396
After treatments	2.0 (1.0, 3.0)	2.0 (1.0, 2.0)	2.0 (2.0, 2.8)	*H* = 3.161	0.206
*p* value^a^	< 0.001	< 0.001	< 0.001		
Color					
Before treatment	7.0 (5.0, 9.0)	7.0 (5.8, 9.0)	8.0 (7.0, 10.0)	*H* = 5.260	0.072
After treatments	4.0 (3.0, 5.0)	3.0 (2.0, 4.0)*	3.0 (3.0, 4.0)	*H* = 8.190	0.017
*p* value^a^	< 0.001	< 0.001	< 0.001		
Stiffness					
Before treatment	6.0 (5.0, 8.0)	7.0 (6.0, 8.0)	7.5 (6.3, 8.8)	*H* = 2.951	0.229
After treatments	3.0 (3.0, 5.0)	3.0 (2.0, 3.0)*	3.0 (2.0, 3.0)*	*H* = 10.243	0.006
*p* value^a^	< 0.001	< 0.001	< 0.001		
Thickness					
Before treatment	6.0 (5.0, 9.0)	6.5 (3.8, 8.0)	7.0 (5.0, 9.8)	*H* = 1.229	0.541
After treatments	3.0 (2.0, 4.0)	2.0 (2.0, 3.0)*	2.0 (2.0, 3.0)*	*H* = 11.731	0.003
*p* value^a^	< 0.001	< 0.001	< 0.001		
Irregularity					
Before treatment	7.0 (5.0, 8.0)	6.0 (5.0, 7.3)	7.0 (6.0, 8.0)	*H* = 2.385	0.303
After treatments	4.0 (3.0, 5.0)	3.0 (3.0, 5.0)	3.0 (3.0, 4.8)	*H* = 1.035	0.596
*p* value^a^	< 0.001	< 0.001	< 0.001		
Total					
Before treatment	36.7 ± 11.4	34.8 ± 7.2	39.4 ± 8.8	*F* = 1.042	0.358
After treatments	20.6 ± 8.1	15.3 ± 3.8*	16.3 ± 3.8*	*F* = 5.388	0.007
*p* value^a^	< 0.001	< 0.001	< 0.001		

Abbreviation: PSAS, Patient Scar Assessment Scale.

* Compared to the single therapy group, *p* ≤ 0.05.

^a^ Comparison between the scores of the PSAS before and after treatments.

^b^ Comparison between the scores of the PSAS in different groups.

The VAS used for pain assessment was recorded on the day of treatment and on the first day after treatment, and no statistical difference was found among the three groups (Table 5). However, the pain on the first day after treatment is significantly weaker than that on the day of treatment.

**TABLE 5:** VAS after treatments.

	Single therapy group	Combined therapy Group 1	Combined therapy Group 2	Statistical variables	*p* value^b^
Day 0 after treatment	2.0 (2.0, 3.0)	3.0 (2.0, 4.0)	3.0 (2.0, 4.0)	*H* = 3.112	0.211
Day 1 after treatment	1.0 (0.0, 2.0)	1.0 (0.8, 2.0)	1.0 (0.3, 2.0)	*H* = 0.325	0.850
*p* value^a^	< 0.001	< 0.001	< 0.001		

Abbreviation: VAS, Visual Analog Scale.

^a^ Comparison between the scores of the VAS on the day of treatment and on the first day after treatment.

^b^ Comparison between the scores of the VAS in different groups.

The satisfaction rate of patients in the single therapy group was 88.6%, while both patients in the two combined therapy groups reported a satisfaction rate of 100% (Table 6).

**TABLE 6:** Patient satisfaction with treatment.

	Single therapy group, *n* (%)	Combined therapy Group 1, *n* (%)	Combined therapy Group 2, *n* (%)	Statistical variables	*p* value
Satisfaction				*χ* ^2^ = 4.449	0.332
Satisfied	18 (51.4)	13 (72.2)	14 (70)		
Generally satisfied	13 (37.1)	5 (27.8)	6 (30)		
Dissatisfied	4 (11.4)	0 (0)	0		

The incidence of adverse events was recorded at 22.9% in the single therapy group, 27.9% in the combined therapy Group 1, and 30% in the combined therapy Group 2 with no significant difference observed among the three groups (Table 7). Nevertheless, there were two cases in each of the two combined therapy groups exhibited ecchymosis at the fat donor site. After receiving cold and hot compress treatments, the ecchymosis of all four patients gradually disappeared. Additionally, no severe complications, such as infection and necrosis, were recorded.

**TABLE 7:** Adverse events in patients.

	Single therapy group, *n* (%)	Combined therapy Group 1, *n* (%)	Combined therapy Group 2, *n* (%)	Statistical variables	*p* value
Adverse events				*χ* ^2^ = 5.838	0.699
Erythema	3 (8.6)	1 (5.6)	1 (5.0)		
Pruritus	4 (11.4)	1 (5.6)	2 (10.0)		
Swelling	1 (2.9)	1 (5.6)	1 (5.0)		
Ecchymosis	0 (0)	2 (11.1)	2 (10.0)		
Total	8 (22.9)	5 (27.9)	6 (30.0)		

**FIGURE 4:**
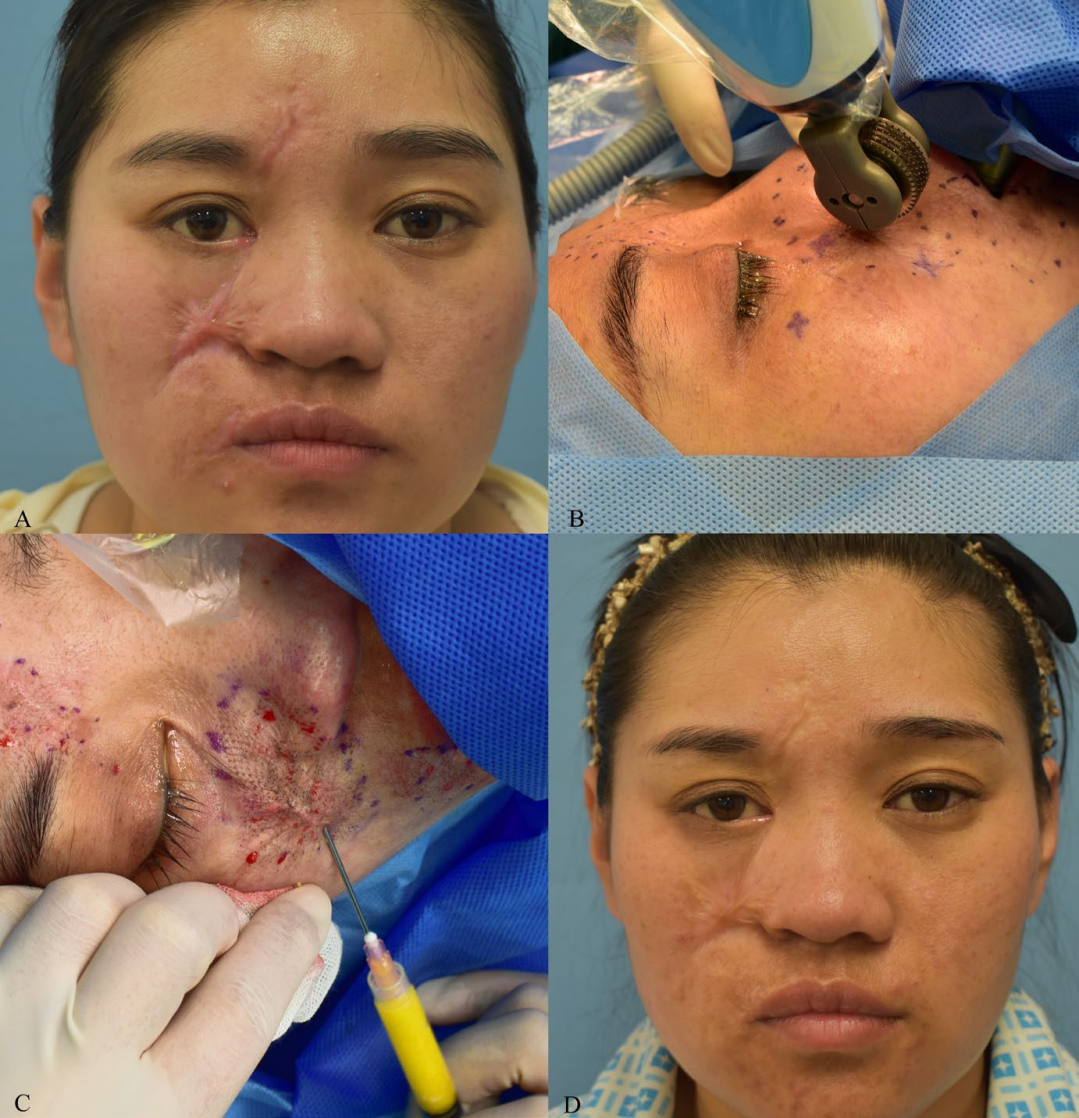
33‐year‐old female. (A) Post traumatic hypertrophic scar. (B) Received micro‐plasma radiofrequency treatment. (C) Received autologous chyle fat grafting treatment. (D) Six months post‐treatment completion.

**FIGURE 5:**
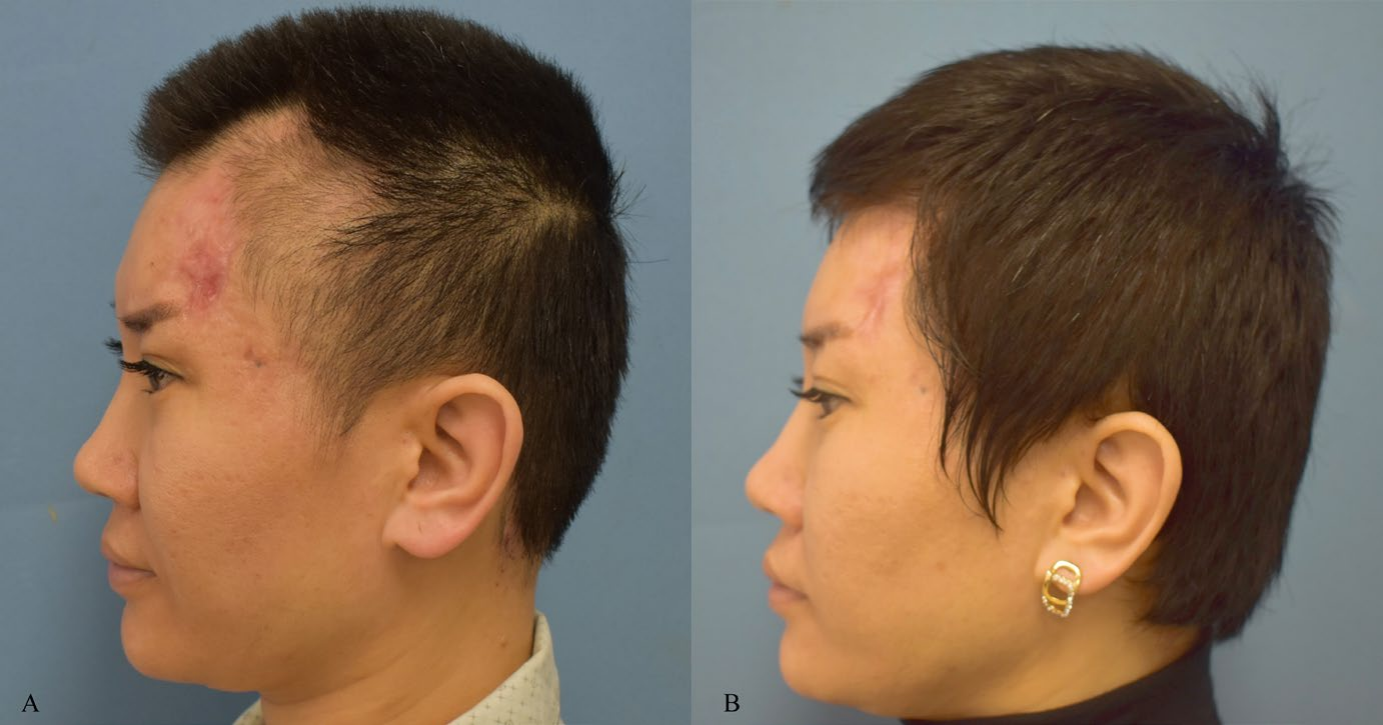
35‐year‐old female. (A) Post traumatic hypertrophic scar. (B) Six months post‐treatment completion.

**FIGURE 6:**
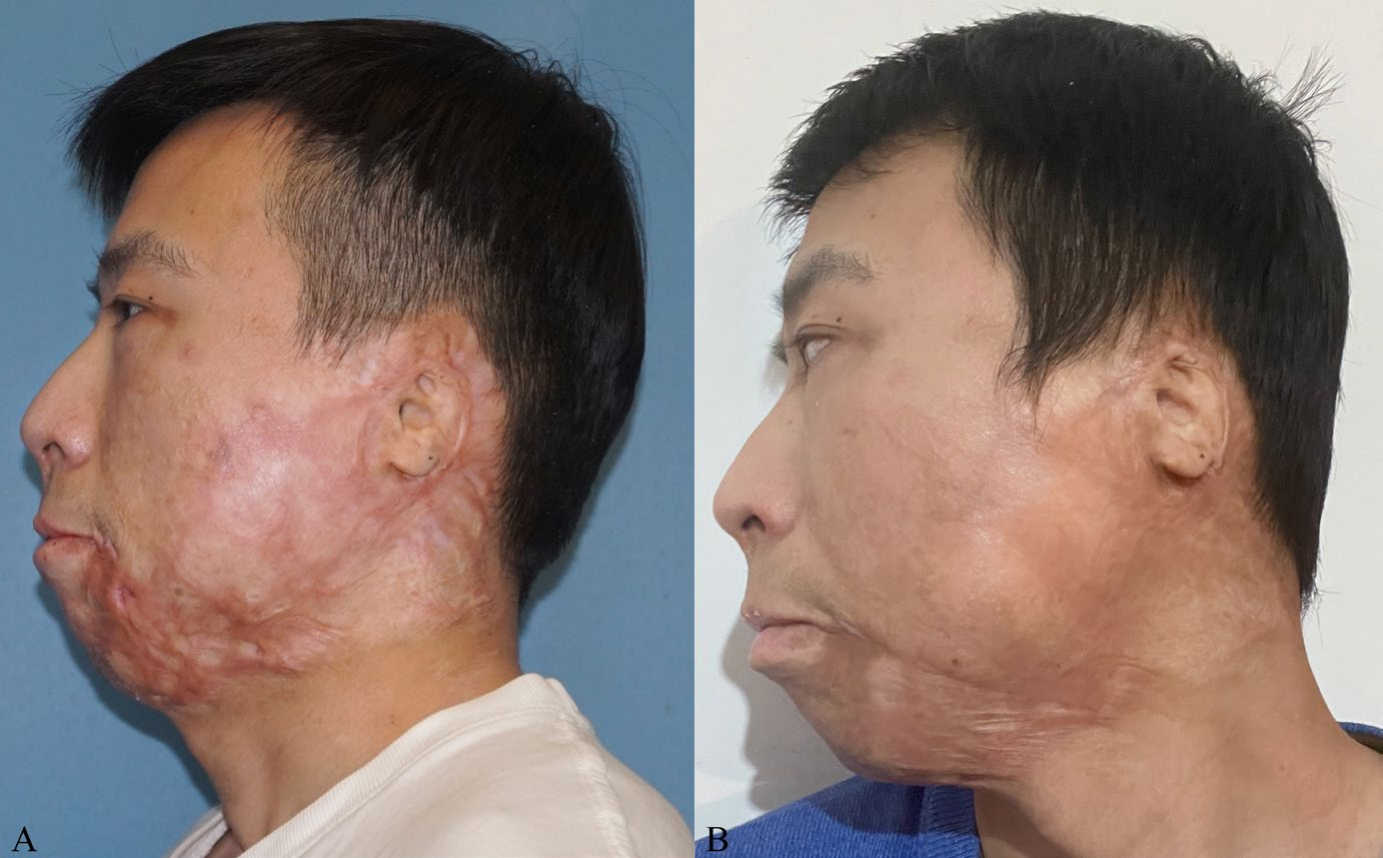
34‐year‐old male. (A) Post burn hypertrophic scar. (B) Six months post‐treatment completion.

Three patients were showcased with pre‐ and post‐treatment photographs in the study to illustrate the effectiveness of MPR when combined with autologous chyle fat grafting for the management of HSs (Figures 4, 5, 6).

## 4 Discussion

HS is a common type of pathological scar observed in clinical practice, with an incidence rate of up to 67% following burns, and even exceeding 75% in non‐white races [16]. Scar tissue forms during the wound healing process whereby excessive proliferation of HS fibroblasts and myofibroblasts occurs in response to injury of the dermis. Consequently, a significant amount of ECM, primarily composed of collagen fibers, is produced accompanied by neovascularization and keratinocyte proliferation, resulting in thickening of the epidermis and dermis [17]. HSs typically manifest around 1–2 months after the injury, commonly in areas of high tension, thereby causing not only cosmetic concerns but also uncomfortable symptoms such as itching and pain.

The pathogenesis HS is intricate and remains incompletely understood. Currently, it is believed that a variety of proteins, cytokines, and signaling pathways are closely related to the formation of HSs, some of which promote fibrosis while others inhibit it. They maintain a balance in healthy tissues, but in HS tissues, this balance is disrupted. Excessive inflammatory cytokines, including tumor necrosis factor‐α (TNF‐α), interleukin‐1β (IL‐1β), and IL‐6, not only promote the proliferation of fibroblasts and synthesis of ECM, but also enhance the production of collagenase inhibitors, leading to the inhibition of collagenase activity [18]. Among these, the TGF‐β (Transforming growth factor‐β)/Smad (Small mothers against decapentaplegic) signaling pathway is considered a key therapeutic target for scar treatment. Previous studies have established the significant involvement of the TGF‐β/Smad signaling pathway in the fibrotic progression of HSs [19, 20]. TGF‐β1 serves as the principal fibrogenic factor, binding to its receptor and initiating the activation of the transcription factor Smad3. Subsequently, this activation facilitates the proliferation of fibroblasts and their transition into myofibroblasts [17, 21]. Myofibroblasts produce ECM and α‐smooth muscle actin (α‐SMA), which are involved in wound contraction and fibrosis. Matrix metalloproteinases (MMPs) are enzymes responsible for degrading ECM. TGF‐β1 has been shown to reduce the MMPs/TIMPs ratio within scar tissues by inhibiting the expression and activity of MMPs, while concurrently promoting the expression of tissue inhibitor of metalloproteinases (TIMPs), leading to the accumulation of ECM [22].

Reactive oxygen species (ROS) are byproducts of oxygen metabolism in organisms, and elevated ROS levels can induce cytotoxic reactions in cells. When the level of oxidative stress in injured tissue surpasses its redox capacity, there is an elevation in the content of ROS within the tissue, consequently extending the duration of the wound healing process and contributing to the formation of HSs [23]. ROS can additionally activate the TGF‐β1/Smad signaling pathway, thereby promoting fibrosis and further stimulating ROS production [24].

The growing focus on regenerative medicine and anti‐aging approaches has led to an increasing interest in fat grafting. Bruno et al. injected fat particles obtained by Coleman technique into burn scars, and found that elastic fibers regenerated in scar tissues, alignment of collagen fibers returned to normal, and clinically found that the patient's aesthetics and function were significantly improved [25]. Autologous fat grafting has many advantages, such as rich source, easy access, less trauma and good biocompatibility, so it is widely used. In recent years, the clinical application of fat grafting has evolved from granular fat filling therapy to chyle fat therapy. Chyle fat is a fat suspension obtained by mechanical emulsification and filtration, which is easier to inject into dense scar tissues than fat particles. Studies have demonstrated that shear stress during mechanical processing can induce upregulation of certain progenitor cell phenotypes, such as mesenchymal stem cells and endothelial progenitor cells, which may explain the powerful regenerative effects of chyle fat grafting and the remarkable clinical results achieved [26]. Jan et al. employed unfiltered nanofat to treat facial postburn scars, resulting in significant improvements in both pigmentation and pliability of the scars [27]. Similarly, Rageh et al. utilized nanofat to inject and observed marked improvements in scar thickness and pliability. Histopathological analysis revealed significant increases in the thickness of the epidermis, collagen fibers, and elastic fibers following nanofat injection [28]. The findings of these studies align with those of our study, demonstrating superior outcomes for patients who underwent autologous chyle fat grafting. In animal experiments, Chen et al. constructed a mouse model of HSs and found that the chyle fat exerts a more potent antifibrotic effect compared to triamcinolone. This was supported by a pronounced reduction in fibroblasts count and collagen fibers content, as well as an elevated level of decorin, a known anti‐fibrotic factor [29].

The mechanism of the treatment of HSs by chyle fat remains partially understood, and current consensus suggests that ADSCs play a pivotal role [30]. In culturing of chyle fat, very few adipocytes were observed and the predominant components were ADSCs and ECM [5, 8]. ADSCs are stem cells derived from adipose tissue with multiple differentiation potential, many researchers are interested in its application in the treatment of HSs. In 2001, Zuk et al. isolated ADSCs from human fat obtained by liposuction and found that it was easier to be separated and cultured than other mesenchymal stem cells [31]. After intradermal injection of ADSCs into the HSs on rabbit ears, Zhang et al. observed a significant inhibition of gene expression of α‐SMA and collagen I. This inhibition led to the improvement of both collagen deposition and alignment in the HSs [32]. Maria et al. reported a significant decrease in the expression of TNF‐α and IL‐1β in the skin following treatment with ADSCs [33]. In the experiment by Li et al., they demonstrated that ADSCs down‐regulate the expression of nuclear factor erythroid‐2‐related factor 2 (Nrf2) in HS fibroblasts, resulting in decreased expression of antioxidant enzymes including heme oxygenase 1 and accumulation of intracellular ROS. As a result, ADSCs promote apoptosis in HS fibroblasts while simultaneously inhibiting their proliferation and migration [34]. Nrf2 is the core anti‐oxidative stress signaling protein found in current studies [35].

Currently, it is widely accepted that ADSCs inhibit scar formation by paracrine mechanism of releasing cytokines, chemokines or growth factors in the surrounding environment. Chen et al. proved that chyle fat–derived stem cells belong to a subcategory of ADSCs and reported that application of chyle fat‐derived stem cells conditioned medium inhibited HS fibroblast proliferation, migration, and ECM protein expression, which indicates that ADSCs inhibit fibrosis through paracrine effects [36]. Multiple studies have consistently demonstrated that the treatment of fibrosis with ADSCs leads to a noteworthy reduction in the expression of TGF‐β1, highlighting its crucial impact. In a study conducted by Spiekman et al., it was found that the ADSCs conditioned medium inhibited the TGF‐β1‐induced proliferation of dermal fibroblasts and reduced their contractility, while also stimulating the expression of MMPs [37]. Following co‐culture with ADSCs, there was a significant decrease in the expression levels of collagen I, collagen III, TGF‐β1, IL‐6, and α‐SMA in HS fibroblasts. Additionally, there was a significant increase in the expression levels of decorin and the MMPs/TIMPs ratio in HS fibroblasts [38]. ADSCs inhibit the differentiation of fibroblasts into myofibroblasts by down‐regulating TGF‐β1 through the secretion of hepatocyte growth factor (HGF) and IL‐10 [39, 40].

MPR is a novel technology different from the traditional photoelectric therapies, characterizing with both ablation and thermal effect, which has the advantages of exact effectiveness, fewer adverse effects and shorter recovery time. During treatment, MPR does not vaporize tissue, leaving a layer of intact and dry epidermis to act as a natural biological dressing to promote faster recovery [41]. Previous studies have reported that it is safe and effective in the treatment of acne scars and the hyperpigmentation, thickness, and flexibility of non‐hypertrophic burn scars [42, 43, 44]. In a rabbit model, Zhang et al. demonstrated the application of MPR for HSs resulted in reduced micro‐vessel and fibroblast density within the scar tissue and rearranged collagen fibers by decreasing the levels of IL‐8 and macrophage chemotactic protein‐1 (MCP‐1) [45].

Numerous clinical studies have investigated the effectiveness of MPR, both as a single treatment and in combination with other therapies, for HSs. MPR has demonstrated positive effects in the treatment of HSs by stimulating the remodeling of collagen fibers [11, 46]. To enhance treatment effectiveness, some researchers opt for a combination of MPR with a diverse array of nonsurgical therapies. Combining MPR with glucocorticoid injections not only led to a significant improvement in the color and texture of postburn HSs, but also effectively reduced local pain and itching [47]. Furthermore, multiple sessions of MPR combined with ablative fractional carbon dioxide laser treatment for HSs had proven to be effective without increasing the incidence of adverse reactions [48]. Histological studies showed that MPR creates superficial and broad “crater” shaped microchannels, while the fractional carbon dioxide laser created narrow and deep “cone” shaped microchannels [49].

This study is the first, to the best of our knowledge, to concurrently utilize autologous chyle fat grafting combined with MPR for the treatment of HSs. The study findings validate that combining these two therapies outperformed MPR alone in the treatment of HSs, especially in terms of improvements in height, vascularity, and pliability. Additionally, we found that the combined therapy, whether administered before or after the application of MPR alone, did not affect the therapeutic outcomes. In terms of safety, the combined therapy did not result in a higher overall incidence of adverse events. However, it is important to emphasize the necessity of adhering to standardized procedures during both the fat extraction and injection processes to minimize the risk of ecchymosis at the donor site and alleviate post‐treatment pain at the recipient site.

Why did patients in the combined therapy group receive MPR treatment before autologous chyle fat grafting treatment? We believe there are several reasons. This sequence ensures the avoidance of thermal damage to the chyle fat caused by the heat generated during MPR treatment. Following MPR treatment, the collagen fiber in the scar tissues undergoes remodeling, resulting in a loose tissue structure that facilitates the injection of chyle fat at the treatment site. Additionally, MPR reduces the number and density of micro‐vessels, thereby minimizing the risk of vascular embolism during chyle fat injection.

Several limitations of this study should be acknowledged. First, it is important to note that this study is a retrospective clinical study conducted at a single center and the sample size may not be sufficiently large, which may have implications on the generalizability of the findings. Second, the follow‐up duration after treatment was limited to 6 months, which may not provide adequate time to fully observe and evaluate the treatment effects. In the future, we aim to undertake a prospective study involving a larger population, with a longer follow‐up duration, and conducted across multiple centers to address these limitations and enhance the robustness of our findings.

## 5 Conclusion

This study revealed the combination of MPR and autologous chyle fat grafting showed superior effectiveness compared to MPR alone in treating HSs, without any observed increase in overall adverse event frequency. For patients diagnosed with HS, this combination therapy stands as a promising and effective clinical intervention.

## Author Contributions

Peixuan Zhang contributed to the design of the study, performed statistical analyses, and drafted the manuscript. Haina Pei contributed to the design of the study and acquisition and interpretation of data. Guiwen Zhou and Qiang Fu contributed to the literature search. Ruiqi Bai, Pianpian Lin, Qian Wu contributed to the data collection. Xiao Xu and Minliang Chen contributed to the design of the study and critically revised the manuscript.

## Ethics Statement

The study was approved by the Ethics Committee of the Fourth Medical Center of Chinese People's Liberation Army General Hospital (No. 2023KY127‐HS001). The consent of clinical data and photos for research purposes was obtained from the patients.

## Conflicts of Interest

All authors declare no conflicts of interest.

## Data Availability

All relevant data are within the manuscript and its additional files.

## References

[1] M. Rodrigues , N. Kosaric , C. A. Bonham , and G. C. Gurtner , “Wound Healing: A Cellular Perspective,” Physiological Reviews 99, no. 1 (2019): 665–706, 10.1152/physrev.00067.2017. 30475656 PMC6442927

[2] H. Lee and Y. Jang , “Recent Understandings of Biology, Prophylaxis and Treatment Strategies for Hypertrophic Scars and Keloids,” International Journal of Molecular Sciences 19, no. 3 (2018): 711, 10.3390/ijms19030711. 29498630 PMC5877572

[3] Z.‐C. Wang , W.‐Y. Zhao , Y. Cao , et al., “The Roles of Inflammation in Keloid and Hypertrophic Scars,” Frontiers in Immunology 11 (2020): 603187, 10.3389/fimmu.2020.603187. 33343575 PMC7746641

[4] R. Ogawa , “The Most Current Algorithms for the Treatment and Prevention of Hypertrophic Scars and Keloids: A 2020 Update of the Algorithms Published 10 Years Ago,” Plastic and Reconstructive Surgery 149, no. 1 (2021): 79e–94e, 10.1097/prs.0000000000008667. PMC8687618 34813576

[5] P. Tonnard , A. Verpaele , G. Peeters , M. Hamdi , M. Cornelissen , and H. Declercq , “Nanofat Grafting,” Plastic and Reconstructive Surgery 132, no. 4 (2013): 1017–1026, 10.1097/PRS.0b013e31829fe1b0. 23783059

[6] E. Behrangi , S. Moradi , M. Ghassemi , et al., “The Investigation of the Efficacy and Safety of Stromal Vascular Fraction in the Treatment of Nanofat‐Treated Acne Scar: A Randomized Blinded Controlled Clinical Trial,” Stem Cell Research & Therapy 13, no. 1 (2022): 298, 10.1186/s13287-022-02957-2. 35841057 PMC9284502

[7] S. Uyulmaz , N. Sanchez Macedo , F. Rezaeian , P. Giovanoli , and N. Lindenblatt , “Nanofat Grafting for Scar Treatment and Skin Quality Improvement,” Aesthetic Surgery Journal 38, no. 4 (2018): 421–428, 10.1093/asj/sjx183. 29365061

[8] X. Xu , L. Lai , X. Zhang , et al., “Autologous Chyle Fat Grafting for the Treatment of Hypertrophic Scars and Scar‐Related Conditions,” Stem Cell Research & Therapy 9, no. 1 (2018): 64, 10.1186/s13287-018-0782-8. 29523181 PMC5845268

[9] S. Yu and H. Li , “Microplasma Radiofrequency Technology Combined With Triamcinolone Improved the Therapeutic Effect on Chinese Patients With Hypertrophic Scar and Reduced the Risk of Tissue Atrophy,” Therapeutics and Clinical Risk Management 10, no. 12 (2016): 743–747, 10.2147/tcrm.S104109. PMC4869595 27274259

[10] S. Halachmi , A. Orenstein , T. Meneghel , and M. Lapidoth , “A Novel Fractional Micro‐Plasma Radio‐Frequency Technology for the Treatment of Facial Scars and Rhytids: A Pilot Study,” Journal of Cosmetic and Laser Therapy 12, no. 5 (2010): 208–212, 10.3109/14764172.2010.514921. 20825258 PMC2956449

[11] A. Baroni and P. Verolino , “Plasma Radiofrequency Ablation for Scar Treatment,” Journal of Clinical Medicine 11, no. 1 (2021): 140, 10.3390/jcm11010140. 35011879 PMC8745684

[12] S. Coleman , “Long‐Term Survival of Fat Transplants: Controlled Demonstrations,” Aesthetic Plastic Surgery 19, no. 5 (1995): 421–425, 10.1007/BF00453875. 8526158

[13] M. Baryza and G. Baryza , “The Vancouver Scar Scale: An Administration Tool and Its Interrater Reliability,” Journal of Burn Care & Rehabilitation 16, no. 5 (1995): 535–538, 10.1097/00004630-199509000-00013. 8537427

[14] A. L. van de Kar , L. U. M. Corion , M. J. C. Smeulders , L. J. Draaijers , C. M. A. M. van der Horst , and P. P. M. van Zuijlen , “Reliable and Feasible Evaluation of Linear Scars by the Patient and Observer Scar Assessment Scale,” Plastic and Reconstructive Surgery 116, no. 2 (2005): 514–522, 10.1097/01.prs.0000172982.43599.d6. 16079683

[15] T. Kono , W. F. Groff , H. Sakurai , T. Yamaki , K. Soejima , and M. Nozaki , “Treatment of Traumatic Scars Using Plasma Skin Regeneration (PSR) System,” Lasers in Surgery and Medicine 41, no. 2 (2009): 128–130, 10.1002/lsm.20723. 19226574

[16] K. M. Bombaro , L. H. Engrav , G. J. Carrougher , et al., “What Is the Prevalence of Hypertrophic Scarring Following Burns?,” Burns 29, no. 4 (2003): 299–302, 10.1016/s0305-4179(03)00067-6. 12781605

[17] T. Zhang , X.‐F. Wang , Z.‐C. Wang , et al., “Current Potential Therapeutic Strategies Targeting the TGF‐β/Smad Signaling Pathway to Attenuate Keloid and Hypertrophic Scar Formation,” Biomedicine & Pharmacotherapy 129 (2020): 110287, 10.1016/j.biopha.2020.110287. 32540643

[18] K. Hayashida , S. Yamakawa , and E. Shirakami , “Strategies to Prevent Hypertrophic Scar Formation: A Review of Therapeutic Interventions Based on Molecular Evidence,” Burns Trauma 8 (2020): tkz003, 10.1093/burnst/tkz003. 32341924 PMC7175766

[19] X. Nong , G. Rajbanshi , L. Chen , et al., “Effect of Artesunate and Relation With TGF‐β1 and SMAD3 Signaling on Experimental Hypertrophic Scar Model in Rabbit Ear,” Archives of Dermatological Research 311, no. 10 (2019): 761–772, 10.1007/s00403-019-01960-7. 31396694 PMC6815271

[20] Y. Huang , H. Zhao , Y. Zhang , et al., “Enhancement of Zyxin Promotes Skin Fibrosis by Regulating FAK/PI3K/AKT and TGF‐β Signaling Pathways via Integrins,” International Journal of Biological Sciences 19, no. 8 (2023): 2394–2408, 10.7150/ijbs.77649. 37215989 PMC10197900

[21] J. Penn , A. Grobbelaar , and K. Rolfe , “The Role of the TGF‐β Family in Wound Healing, Burns and Scarring: A Review,” International Journal of Burns and Trauma 2, no. 1 (2012): 18–28. 22928164 PMC3415964

[22] K. W. Finnson , S. McLean , G. M. di Guglielmo , and A. Philip , “Dynamics of Transforming Growth Factor Beta Signaling in Wound Healing and Scarring,” Advances in Wound Care 2, no. 5 (2013): 195–214, 10.1089/wound.2013.0429. 24527343 PMC3857355

[23] S. Ellis , E. J. Lin , and D. Tartar , “Immunology of Wound Healing,” Current Dermatology Reports 7, no. 4 (2018): 350–358, 10.1007/s13671-018-0234-9. 30524911 PMC6244748

[24] K. Richter and T. Kietzmann , “Reactive Oxygen Species and Fibrosis: Further Evidence of a Significant Liaison,” Cell and Tissue Research 365, no. 3 (2016): 591–605, 10.1007/s00441-016-2445-3. 27345301 PMC5010605

[25] A. Bruno , G. Delli Santi , L. Fasciani , M. Cempanari , M. Palombo , and P. Palombo , “Burn Scar Lipofilling: Immunohistochemical and Clinical Outcomes,” Journal of Craniofacial Surgery 24, no. 5 (2013): 1806–1814, 10.1097/SCS.0b013e3182a148b9. 24036785

[26] D. A. Banyard , C. N. Sarantopoulos , A. A. Borovikova , et al., “Phenotypic Analysis of Stromal Vascular Fraction After Mechanical Shear Reveals Stress‐Induced Progenitor Populations,” Plastic and Reconstructive Surgery 138, no. 2 (2016): 237e–247e, 10.1097/prs.0000000000002356. PMC5845841 27465185

[27] S. N. Jan , M. M. Bashir , F. A. Khan , et al., “Unfiltered Nanofat Injections Rejuvenate Postburn Scars of Face,” Annals of Plastic Surgery 82, no. 1 (2019): 28–33, 10.1097/sap.0000000000001631. 30285990

[28] M. A. Rageh , M. El‐Khalawany , and S. M. A. Ibrahim , “Autologous Nanofat Injection in Treatment of Scars: A Clinico‐Histopathological Study,” Journal of Cosmetic Dermatology 20, no. 10 (2021): 3198–3204, 10.1111/jocd.14363. 34357682

[29] J. Chen , L. Lai , K. Ma , et al., “The Effect of Chyle Fat Injection on Human Hypertrophic Scars in an Animal Model,” Annals of Plastic Surgery 82, no. 6 (2019): 622–627, 10.1097/sap.0000000000001784. 30633019

[30] K. T. Putri and T. O. H. Prasetyono , “A Critical Review on the Potential Role of Adipose‐Derived Stem Cells for Future Treatment of Hypertrophic Scars,” Journal of Cosmetic Dermatology 21, no. 5 (2021): 1913–1919, 10.1111/jocd.14385. 34619011

[31] P. Zuk , M. Zhu , H. Mizuno , J. Huang , J. Futrell , and A. Katz , “Multilineage Cells From Human Adipose Tissue: Implications for Cell‐Based Therapies,” Tissue Engineering 7, no. 2 (2001): 211–228, 10.1089/107632701300062859. 11304456

[32] Q. Zhang , L.‐N. Liu , Q. Yong , J.‐C. Deng , and W.‐G. Cao , “Intralesional Injection of Adipose‐Derived Stem Cells Reduces Hypertrophic Scarring in a Rabbit Ear Model,” Stem Cell Research & Therapy 6, no. 1 (2015): 145, 10.1186/s13287-015-0133-y. 26282394 PMC4539671

[33] A. T. J. Maria , K. Toupet , M. Maumus , et al., “Human Adipose Mesenchymal Stem Cells as Potent Anti‐Fibrosis Therapy for Systemic Sclerosis,” Journal of Autoimmunity 70 (2016): 31–39, 10.1016/j.jaut.2016.03.013. 27052182

[34] S. Li , J. Yang , J. Sun , and M. Chen , “Adipose‐Derived Mesenchymal Stem Cells Alleviate Hypertrophic Scar by Inhibiting Bioactivity and Inducing Apoptosis in Hypertrophic Scar Fibroblasts,” Cells 11, no. 24 (2022): 4024, 10.3390/cells11244024. 36552789 PMC9776926

[35] S. K. Niture and A. K. Jaiswal , “Nrf2 Protein Up‐Regulates Antiapoptotic Protein Bcl‐2 and Prevents Cellular Apoptosis,” Journal of Biological Chemistry 287, no. 13 (2012): 9873–9886, 10.1074/jbc.M111.312694. 22275372 PMC3323009

[36] J. Chen , Z. Li , Z. Huang , L. Liang , and M. Chen , “Chyle Fat–Derived Stem Cells Conditioned Medium Inhibits Hypertrophic Scar Fibroblast Activity,” Annals of Plastic Surgery 83, no. 3 (2019): 271–277, 10.1097/sap.0000000000001932. 31149905

[37] M. Spiekman , E. Przybyt , J. A. Plantinga , S. Gibbs , B. van der Lei , and M. C. Harmsen , “Adipose Tissue–Derived Stromal Cells Inhibit TGF‐β1–Induced Differentiation of Human Dermal Fibroblasts and Keloid Scar–Derived Fibroblasts in a Paracrine Fashion,” Plastic and Reconstructive Surgery 134, no. 4 (2014): 699–712, 10.1097/prs.0000000000000504. 25357030

[38] J. Deng , Y. Shi , Z. Gao , et al., “Inhibition of Pathological Phenotype of Hypertrophic Scar Fibroblasts via Coculture With Adipose‐Derived Stem Cells,” Tissue Engineering Part A 24, no. 5–6 (2018): 382–393, 10.1089/ten.tea.2016.0550. 28562226

[39] A. A. Borovikova , M. E. Ziegler , D. A. Banyard , et al., “Adipose‐Derived Tissue in the Treatment of Dermal Fibrosis,” Annals of Plastic Surgery 80, no. 3 (2018): 297–307, 10.1097/sap.0000000000001278. 29309331

[40] A. Ejaz , M. W. Epperly , W. Hou , J. S. Greenberger , and J. P. Rubin , “Adipose‐Derived Stem Cell Therapy Ameliorates Ionizing Irradiation Fibrosis via Hepatocyte Growth Factor‐Mediated Transforming Growth Factor‐β Downregulation and Recruitment of Bone Marrow Cells,” Stem Cells 37, no. 6 (2019): 791–802, 10.1002/stem.3000. 30861238 PMC8457883

[41] E. V. di Brizzi , T. Russo , M. Agozzino , et al., “Plasma Radiofrequency Ablation for Treatment of Benign Skin Lesions: Clinical and Reflectance Confocal Microscopy Outcomes,” Skin Research and Technology 25, no. 6 (2019): 773–776, 10.1111/srt.12716. 31111566

[42] T. Lan , Y. Xiao , L. Tang , M. R. Hamblin , and R. Yin , “Treatment of Atrophic Acne Scarring With Fractional Micro‐Plasma Radio‐Frequency in Chinese Patients: A Prospective Study,” Lasers in Surgery and Medicine 50, no. 8 (2018): 844–850, 10.1002/lsm.22825. 29663460 PMC6146052

[43] L.‐Z. Wang , J.‐P. Ding , M.‐Y. Yang , D.‐W. Chen , and B. Chen , “Treatment of Facial Post‐Burn Hyperpigmentation Using Micro‐Plasma Radiofrequency Technology,” Lasers in Medical Science 30, no. 1 (2014): 241–245, 10.1007/s10103-014-1649-6. 25209007

[44] S. Wang , J. Mi , Q. Li , R. Jin , and J. Dong , “Fractional Microplasma Radiofrequency Technology for Non‐hypertrophic Post‐Burn Scars in Asians: A Prospective Study of 95 Patients,” Lasers in Surgery and Medicine 49, no. 6 (2017): 563–569, 10.1002/lsm.22640. 28220505

[45] W. Zhang , X. Cheng , H. Li , and M. Cao , “Evaluation of the Therapeutic Effect of Micro‐Plasma Radio Frequency on Hypertrophic Scars in Rabbit Ears,” Lasers in Medical Science 33, no. 9 (2018): 1961–1968, 10.1007/s10103-018-2562-1. 30003425

[46] N. M. Pinheiro , P. R. Melo , V. O. Crema , and A. C. Mendonça , “Effects of Radiofrequency Procedure on Hypertrophic Scar due to Burns,” Journal of the European Academy of Dermatology and Venereology 29, no. 1 (2014): 187–189, 10.1111/jdv.12388. 24673645

[47] S. X. Qu Ca , J. Hu , S. Zhan , et al., “Clinical Observation of Microplasma Radiofrequency Technology Combined With Glucocorticoid Injection in the Treatment of Hundreds of Cases of Hypertrophic Scar After Early Deep Burn and Scald,” Journal of Craniofacial Surgery 34, no. 2 (2023): 687–690, 10.1097/scs.0000000000009121. 36710391

[48] J. Li , D. Wang , Y. Wang , Y. Du , and S. Yu , “Effectiveness and Safety of Fractional Micro‐Plasma Radio‐Frequency Treatment Combined With Ablative Fractional Carbon Dioxide Laser Treatment for Hypertrophic Scar: A Retrospective Study,” Annals of Palliative Medicine 10, no. 9 (2021): 9800–9809, 10.21037/apm-21-2153. 34628906

[49] M.‐K. Shin , J. H. Choi , S. B. Ahn , and M. H. Lee , “Histologic Comparison of Microscopic Treatment Zones Induced by Fractional Lasers and Radiofrequency,” Journal of Cosmetic and Laser Therapy 16, no. 6 (2014): 317–323, 10.3109/14764172.2014.957216. 25148410

